# A Marine-Derived Steroid from *Rhodococcus* sp., 3,12-Dioxochola-4,6-dien-24-oic Acid, Enhances Skin Re-Epithelialization and Tissue Repair

**DOI:** 10.3390/md23070292

**Published:** 2025-07-19

**Authors:** Mücahit Varlı, Hui Tan, Chaeyoung Lee, Jeongyun Lee, Ji Young Lee, Jeong-Hyeon Kim, Songyi Lee, Hangun Kim, Sang-Jip Nam

**Affiliations:** 1 College of Pharmacy, Sunchon National University, Sunchon 57922, Republic of Korea; mucahitvarli@s.scnu.ac.kr (M.V.); lju00333@naver.com (J.L.); 2 Department of Chemistry and Nanoscience, Ewha Womans University, Seoul 03760, Republic of Korea; 247878kobe@naver.com (H.T.); chaeyoung510@ewha.ac.kr (C.L.); sub03101@gmail.com (J.-H.K.); 3 Institute of Sustainable Earth and Environmental Dynamics (SEED), Pukyong National University, 365 Sinseon-ro, Nam-gu, Busan 48547, Republic of Korea; jji123789@naver.com; 4 Department of Chemistry, Pukyong National University, Busan 48513, Republic of Korea; slee@pknu.ac.kr; 5 Industry 4.0 Convergence Bionics Engineering, Pukyong National University, Busan 48513, Republic of Korea; 6 Graduate Program in Innovative Biomaterials Convergence, Ewha Womans University, Seoul 03760, Republic of Korea

**Keywords:** wound healing, tissue repair, *Rhodococcus* sp., marine drugs, 3,12-dioxochola-4,6-dien-24-oic acid, glucocorticoid receptor

## Abstract

The discovery of bioactive natural compounds from microbes holds promise for regenerative medicine. In this study, we identified and characterized a steroid-like compound, 3,12-dioxochola-4,6-dien-24-oic acid (DOCDA), from a crude extract of *Rhodococcus* sp. DOCDA significantly promoted wound healing by enhancing HaCaT cell invasion and migration. It upregulated key growth factors (EGF, VEGF-A, IGF, TGF-*β*, and HGF), indicating the activation of regenerative signaling. Additionally, DOCDA increased the expression of genes related to focal adhesion and cytoskeletal regulation (ITGB1, ITGA4, FAK, SRC, RHOA, CDC42, RAC1, and paxillin), supporting enhanced cellular motility and remodeling. Notably, DOCDA promoted stem-like properties in HaCaT cells, as shown by increased spheroid formation and elevated levels of the stemness markers ALDH1 and CD44. Target prediction and molecular docking identified the glucocorticoid receptor (GR) as the primary target of DOCDA, with a docking score of −7.7 kcal/mol. Network and pathway enrichment analysis revealed that GR-linked pathways were significantly associated with wound healing, including steroid hormone signaling, inflammation, immune responses, and cell migration. In vivo, the topical application of DOCDA led to over 70% wound closure in mice by day 5. These findings suggest that DOCDA is a steroid-like compound that accelerates wound healing and may serve as a potential agent in regenerative therapy.

## 1. Introduction

Skin injuries are a common and significant health concern worldwide, resulting from various causes such as traumatic wounds, surgical procedures, chronic conditions like diabetes, and side effects of treatments, including radiotherapy. These diverse factors can impair the wound healing process, leading to delayed recovery, increased susceptibility to infection, and a substantial burden on healthcare systems [1,2,3,4]. Skin wound healing is a vital physiological process that restores the integrity and function of the skin after injury. The process involves a highly coordinated sequence of cellular and molecular events to repair damaged tissue and maintain the body’s protective barrier [5]. Recent advances in regenerative medicine have highlighted the importance of targeting key biological processes involved in wound repair. These include focal adhesion dynamics, which govern cell migration and tissue remodeling; stem cell activation and maintenance, which support regeneration and long-term repair; and growth factor signaling, which regulates cellular proliferation, differentiation, and angiogenesis [4,6,7,8,9,10,11,12,13]. Therefore, a deeper understanding of these mechanisms is essential for the development of new and effective therapies to address this widespread clinical challenge.

The glucocorticoid receptor (GR) is a nuclear receptor that mediates the effects of steroid hormones, particularly glucocorticoids, at the cellular level. GR plays a critical role in numerous physiological processes, including immune regulation, metabolism, and stress responses. Wound healing is a complex, multi-phase process encompassing inflammation, proliferation, and remodeling, and GR signaling is known to influence each of these stages through both genomic and non-genomic mechanisms [14,15,16,17,18]. Cortisol, an endogenously secreted glucocorticoid, is involved in stress responses, immune modulation, and metabolic balance [19]. Clinically, corticosteroid-based drugs are widely used, especially for treating inflammatory diseases. However, prolonged or high-dose use of these steroids can adversely affect wound healing by suppressing epithelialization and fibroblast activity, ultimately delaying tissue repair [2,20,21]. Given these limitations, recent research has focused on the development and use of selective glucocorticoid receptor modulators (SEGRMs) as alternative therapeutic agents. These compounds aim to retain the anti-inflammatory benefits of conventional corticosteroids while minimizing their adverse effects [22,23,24]. Thus, modulating GR activity presents a promising strategy for managing chronic or non-healing wounds, offering potential improvements in wound care and tissue regeneration.

The wound healing potential of various naturally occurring compounds has been well documented, paving the way for the discovery of therapeutic agents derived from natural sources, including marine-derived bacteria [5,25,26,27] such as the *Rhodococcus* species investigated in this study. *Rhodococcus* is a genus of Gram-positive, aerobic, non-motile bacteria belonging to the order Actinomycetales [28,29]. These bacteria are widely distributed in soil, water, and contaminated environments and are renowned for their metabolic versatility and ability to produce a broad spectrum of bioactive natural products, including antibiotics, steroids, and other biologically active compounds [30,31]. These metabolites have significant applications across medicine, agriculture, and industry [32,33].

In natural product research, *Rhodococcus* strains have demonstrated the capacity to catalyze a variety of biochemical transformations, such as the oxidation, reduction, hydroxylation, ring cleavage, and demethylation of structurally complex molecules [34,35,36]. Their high tolerance to toxic intermediates and robust metabolic systems make them promising candidates for biotransformation, structural modification, and the development of environmentally friendly biocatalytic processes [30,37,38]. However, despite these promising capabilities, reports on the use of *Rhodococcus* strains for natural product isolation remain limited and warrant further exploration. In this study, we isolated 3,12-dioxochola-4,6-dien-24-oic acid (DOCDA) from a marine-derived *Rhodococcus* sp. and investigated its potential effects on wound healing. To our knowledge, this is the first study to evaluate the therapeutic potential of DOCDA, a previously uncharacterized natural compound, in the context of wound repair.

## 2. Results and Discussion

### 2.1. Structural Determination

**Figure 1:**
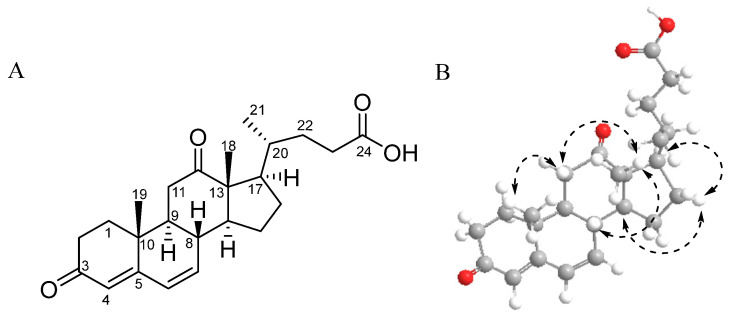
Chemical structure (**A**) and key NOESY correlations (**B**) of DOCDA.

DOCDA (Figure 1A) was isolated as a dark yellow oil, and LR-ESI-MS spectroscopic analysis revealed an ion peak at m/z 385.23 [M + H]^+^. The ^1^H NMR spectrum of DOCDA displayed three olefinic protons at *δ*_H_ 6.22 (dd, *J* = 9.9, 2.5 Hz, H-6), 6.19 (dd, *J* = 9.9, 2.5 Hz, H-7), and 5.66 (s, H-4), two singlet methyl protons at *δ*_H_ 1.15 (s, H-19) and 1.08 (s, H-18), and one doublet methyl proton at *δ*_H_ 0.78 (d, *J* = 6.4 Hz, H-21). The ^13^C and HSQC NMR spectra indicated the presence of twenty-four carbons, including three carbonyl carbons at *δ*_C_ 174.7 (C-24), *δ*_C_ 197.9 (C-3), and *δ*_C_ 212.2 (C-12), four olefin carbon resonances at *δ*_C_ 123.5 (C-4), *δ*_C_ 128.1 (C-6), *δ*_C_ 139.5 (C-7), and *δ*_C_ 162.1 (C-5), three methyl carbons at *δ*_C_ 11.1 (C-18), *δ*_C_ 15.6 (C-19), and *δ*_C_ 18.7 (C-21), and two quaternary carbons at *δ*_C_ 57.2 and *δ*_C_ 36.3. A pregna-4,6-diene-3,12-dione moiety was identified from the ^1^H-^1^H COSY correlations [H-1/H-2/H-3/H-4, H-6/H-7/H-8, H-8/H-10/H-11, H-14/H-15/H-16/H-17] and HMBC correlations [H-4/C-3/C-6/C-10; H-6/C-5/C-7; H-7/C-9/C-14; H-15/C-8/C-14; H-16/C-13/C-20; H-18/C-12/C-13/C-14/C-20; H-19/C-1/C-5/C-9/C-10] [39]. The side chain of DOCDA was elucidated through additional ^1^H-^1^H COSY correlations and HMBC correlations. COSY correlations were observed between H-20/H-21, H-20/H-22, and H-22/H-23. In addition, key HMBC correlations included those from the doublet methyl proton H-21 to C-17 and C-22, and from the methylene proton H-23 to C-20, C-22, and the carbonyl carbon C-24 at *δ*_C_ 174.7. Based on a comparison with previously reported MS data, the compound was identified as DOCDA [40]. The relative stereochemistry of DOCDA was assigned on the basis of a 2D NOESY experiment (Figure 1B). The observation of NOE correlations [H_3_-18/H-11*β*/H-8, H_3_-19/H-11*β*] indicated that both H-8 and H-19 were in *β*-orientation. Additionally, NOE correlations [H-17/H-16α/H-14] established the *α*-orientations of H-14 and H-17. Since almost all 20-methylsterols isolated from natural products exhibit a 20*S* configuration according to the literature [41,42,43], DOCDA was, thus, assigned the same configuration. DOCDA was initially described as a minor unsaturated metabolite produced during the anaerobic degradation of cholic acid by *Pseudomonas* sp., and was later isolated from the plant *Allardia tridactylites* [40,44]. However, the full NMR spectroscopic data for this compound have not yet been reported (Figures S1–S6). To the best of our knowledge, no steroids have been previously isolated from *Rhodococcus* sp. strains. Reported studies have only described the biotransformation of externally supplied steroidal substrates by *Rhodococcus* species [34,45].

### 2.2. Bioactivity Assay

**Figure 2:**
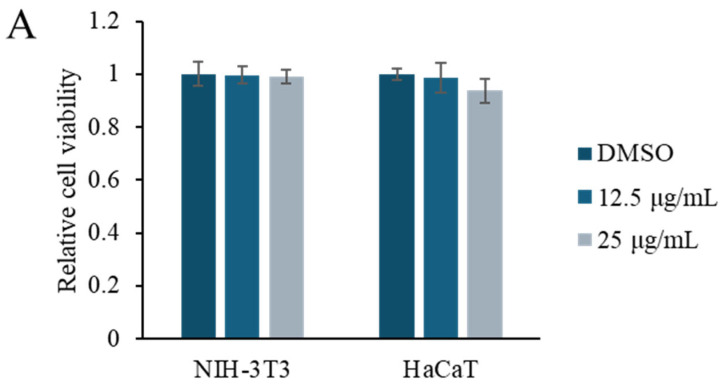

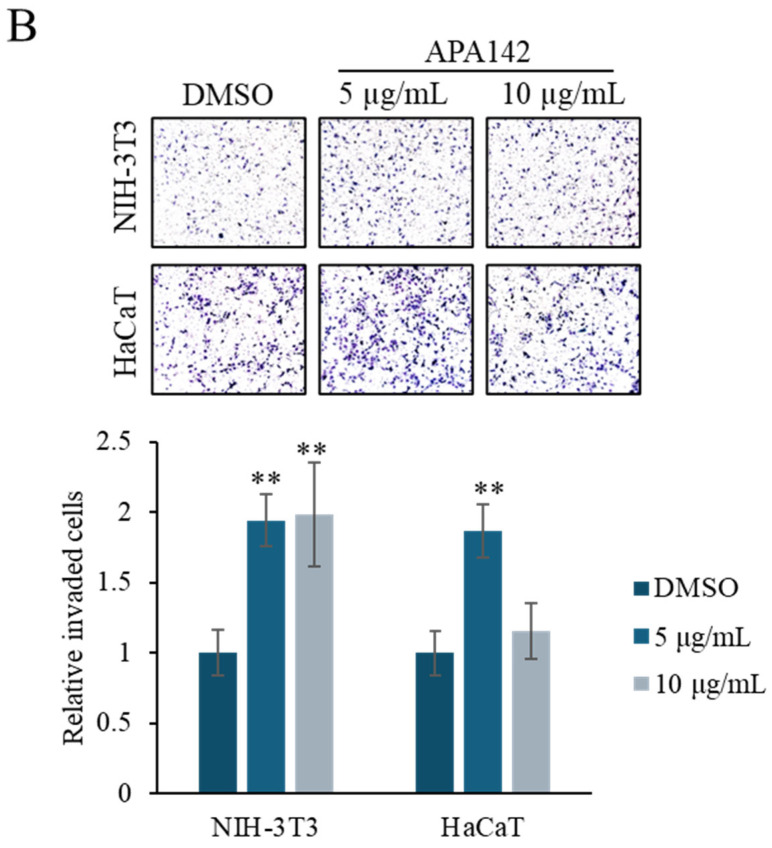
Effect of the *Rhodococcus* sp. crude extract on the cell viability and cell motility. (**A**) MTT assay results showing the viability of NIH-3T3 and HaCaT cells after treatment with *Rhodococcus* sp. crude extract at concentrations of 12.5 and 25 μg/mL for 48 h. Data are presented as mean ± SD (*n* = 6). (**B**) Representative images from the Transwell invasion assay of NIH-3T3 and HaCaT cells treated with 5 and 10 μg/mL of the crude extract. Quantification of invaded cells is shown in the accompanying graph. Data are presented as mean ± SD (*n* = 5). ** *p*  <  0.01 vs. vehicle control (1% DMSO).

NIH-3T3 (mouse embryonic fibroblast) and HaCaT (human keratinocyte) cell lines are frequently preferred for the in vitro modeling of skin biology, wound healing, and cell-to-cell interactions. NIH-3T3 cells are fibroblast-derived and are widely used in the study of events such as extracellular matrix production, growth factor signaling, and the epithelial–mesenchymal transition. On the other hand, HaCaT cells are immortalized keratinocytes derived from the human epidermis and offer a suitable model to evaluate epidermal physiology and dermatological effects, as they largely retain their differentiation abilities. Therefore, we used NIH-3T3 and HaCaT cells. The viability of NIH-3T3 (mouse embryonic fibroblast) and HaCaT (human keratinocyte) cells in response to *Rhodococcus* sp. crude extract was evaluated at concentrations of 12.5 and 25 μg/mL using the methyl thiazolyl tetrazolium (MTT) assay (Figure 2A). No significant changes in viability were observed at either concentration, indicating a low cytotoxicity. To investigate the effect of the crude extract on cell motility, Transwell invasion assays were performed using NIH-3T3 and HaCaT cells treated with 5 or 10 μg/mL of the extract. Treatment with *Rhodococcus* sp. crude extract notably enhanced cell invasion in both cell lines, resulting in an approximately two-fold increase compared to control conditions (Figure 2B).

**Figure 3:**
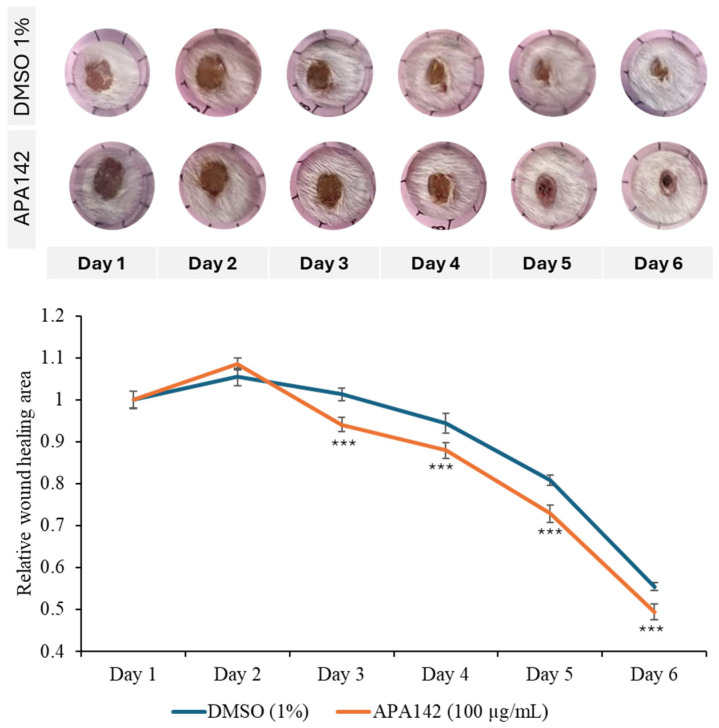
In vivo wound healing assay demonstrating the pro-regenerative effect of *Rhodococcus* sp. crude extract. Representative images of mouse wounds treated topically with either 1% DMSO (control) or *Rhodococcus* sp. crude extract (APA–142, 100 μg/mL) from day 1 to day 6 post-wounding. Quantitative analysis of wound closure rates over the 6-day period is shown, with values normalized to the initial wound area on day 1. Data are presented as mean ± SD (*n* = 6 per group). *** *p*  <  0.001 vs. vehicle control (DMSO).

Additionally, in vivo wound healing models demonstrated that topical application of the crude extract markedly accelerated wound closure, further supporting its pro-regenerative potential (Figure 3). To quantify this effect, wound closure rates were monitored daily over a 6-day period and normalized to the initial wound size recorded on day 1. In the control group treated with 1% DMSO, the wound area slightly increased on day 2 (+5.5%) and day 3 (+1.3%) before gradually decreasing from day 4 onward, reaching approximately 55% of the original wound area still open by day 6. In contrast, wounds treated with APA–142 (100 µg/mL) exhibited a more rapid reduction in wound size beginning after day 3. By day 6, the remaining wound area had decreased to approximately 49% of its initial size, indicating faster wound closure compared to the control. These results suggest a potential pro-regenerative effect of *Rhodococcus* sp., warranting further investigation into its therapeutic applications in processes that benefit from enhanced cell invasion, such as wound healing. Given these promising biological properties, further characterization of the crude extract was undertaken to identify the bioactive component responsible for the observed effects. Bioactivity-guided fractionations of the extract yielded eight fractions, among which the APA–142-C18-F5 fraction selectively promoted cell motility in NIH-3T3 fibroblasts, but did not enhance invasion in the Caco-2 colorectal cancer cell line. This selective activity suggests a targeted pro-wound healing effect (Figure S7). Therefore, the active compound, DOCDA, was isolated from this fraction. 

**Figure 4:**
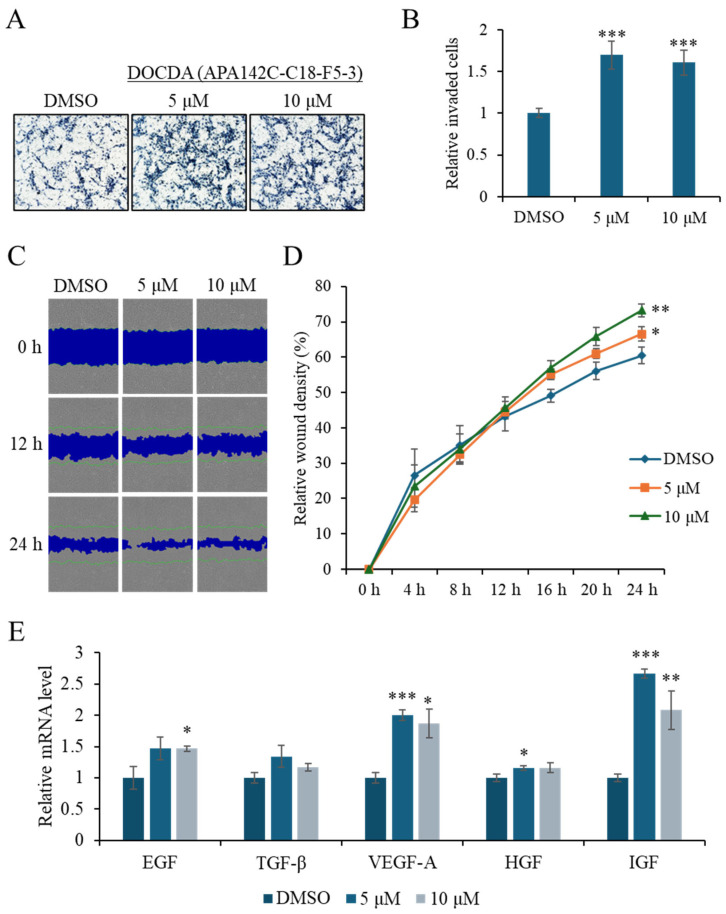
DOCDA enhances invasion, migration, and growth factor expression in HaCaT cells. (**A**,**B**) Transwell invasion assay showing a significant increase in the invasive capacity of HaCaT cells treated with DOCDA compared to the control (DMSO). Cells were treated with DOCDA for 24 h. Quantification of invaded cells is presented as mean ± SD (*n* = 5); *** *p* < 0.001 vs. vehicle (DMSO). (**C**,**D**) Representative images from the scratch wound healing assay at 0, 12 and 24 h demonstrate accelerated wound closure in DOCDA-treated cells. Quantitative analysis of relative wound density (%) reveals a significantly higher wound closure percentage compared to the control group. Data are presented as mean ± SD (*n* = 3); * *p*  <  0.05; ** *p*  <  0.01 vs. vehicle (DMSO). (**E**) qRT-PCR analysis of key growth factors (EGF, TGF-*β*, VEGF-A, HGF, and IGF) in HaCaT cells treated with DOCDA at concentrations of 5 μM and 10 μM. Gene expression levels were normalized to ACTIN and are presented as relative fold change compared to vehicle control (DMSO). Data are shown as mean ± SEM (*n* = 3); * *p*  <  0.05; ** *p*  <  0.01; *** *p* < 0.001 vs. vehicle (DMSO).

To evaluate the effects of DOCDA on wound healing, a series of in vitro assays were conducted, including Transwell invasion assays, and a scratch (wound healing) assay, cell proliferation analysis, and qRT-PCR analysis of key growth factors. In the Transwell invasion assay (Figure 4A,B), DOCDA significantly enhanced the invasive capacity of HaCaT cells. This pro-migratory effect was further confirmed by the scratch wound healing assay, which demonstrated accelerated wound closure in DOCDA-treated cells. Representative images captured at 0, 12 and 24 h (Figure 4C,D) revealed markedly enhanced wound closure in the treatment group. Quantitative analysis showed a significantly higher wound closure rate compared to the control group, reinforcing the positive effect of DOCDA on collective cell motility.

Growth factors play critical roles in skin wound healing by regulating processes such as cell proliferation, inflammation, and angiogenesis [46]. Epidermal growth factor (EGF) accelerates re-epithelialization by promoting keratinocyte proliferation and migration; recent studies have shown that EGF significantly improves healing rates in diabetic foot ulcers [13,47,48]. Transforming growth factor-beta (TGF-*β*) plays a pivotal role in orchestrating the inflammatory response and promotes extracellular matrix (ECM) production. It facilitates the transition from inflammation to proliferation by modulating immune cell recruitment and cytokine secretion, enhancing fibroblast proliferation and differentiation into myofibroblasts, and stimulating the synthesis of ECM components such as collagen and fibronectin. TGF-*β* also contributes to angiogenesis and re-epithelialization, supporting the structural integrity and functional restoration of damaged tissue [12,49]. Vascular endothelial growth factor-A (VEGF-A) promotes angiogenesis by facilitating oxygen and nutrient delivery to the wound site. Combined treatment with modified mRNA forms of VEGF-A and fibroblast growth factor-1 (FGF1) has been shown to markedly improve wound healing in diabetic mouse models [50]. Hepatocyte growth factor (HGF) enhances tissue regeneration by promoting epithelial and endothelial cell migration [11,51], while insulin-like growth factor-1 (IGF-1) accelerates healing by suppressing inflammation and promoting angiogenesis, effects mediated via the Ras/PI3K/IKK/NF-κB signaling pathways [52]. Given the central role of these growth factors in wound healing, we next assessed whether DOCDA modulated their expression in HaCaT cells. Treatment with DOCDA at concentrations of 5 μM and 10 μM significantly upregulated the mRNA expression levels of several key growth factors, including EGF, TGF-*β*, VEGF-A, HGF, and IGF. At the 5 μM dose, EGF, VEGF-A, and IGF levels increased by approximately 1.5- to 2.7-fold compared to the DMSO control group (Figure 4E). Similarly, the expression of these growth factors remained elevated at the 10 μM dose. These findings highlight the potential of DOCDA to promote skin wound healing by enhancing the expression of multiple growth factors involved in regeneration and repair.

**Figure 5:**
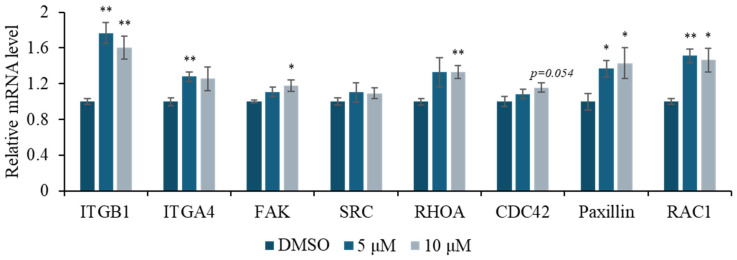
DOCDA treatment enhances expression of focal adhesion and cytoskeletal regulators. Relative mRNA expression levels of key adhesion molecules and signaling mediators, including ITGB1, ITGA4, FAK, SRC, RHOA, CDC42, paxillin, and RAC1, were analyzed by qRT-PCR in HaCaT cells treated with DOCDA at 5 μM and 10 μM for 48 h. Gene expression levels were normalized to ACTIN and are presented as fold changes relative to the vehicle control (DMSO). Data are shown as mean ± SEM (*n* = 3); * *p*  <  0.05; ** *p*  <  0.01 vs. vehicle (DMSO).

DOCDA treatment was also found to enhance the mRNA expression of genes involved in focal adhesion and cell motility in HaCaT cells. Notably, the integrin family members ITGB1 and ITGA4 were significantly upregulated, suggesting increased potential for adhesion to the extracellular matrix [53,54]. Additionally, elevated expressions of the Rho family GTPases RAC1, CDC42, and RHOA, which play pivotal roles in cytoskeletal reorganization [55,56,57], support the involvement of DOCDA in promoting cell migration and morphological adaptation. Focal adhesion kinase (FAK) and SRC family kinases are essential regulators of integrin signaling that coordinate focal adhesion turnover during migration. FAK functions as a central scaffolding and signaling molecule that transmits extracellular signals into intracellular pathways, while SRC facilitates phosphorylation events critical for adhesion dynamics and cytoskeletal remodeling [58,59]. Paxillin, a focal adhesion adaptor protein, interacts with both FAK and SRC to stabilize focal adhesion complexes and support actin cytoskeleton organization, thereby enabling effective migration and adhesion [8,9,10,60]. The DOCDA-induced upregulation of FAK, SRC, and paxillin mRNA levels indicates a stimulatory effect on focal adhesion formation and motility regulation. These findings suggest that DOCDA modulates a network of adhesion- and migration-related genes, thereby activating key mechanisms essential for skin wound healing (Figure 5).

**Figure 6:**
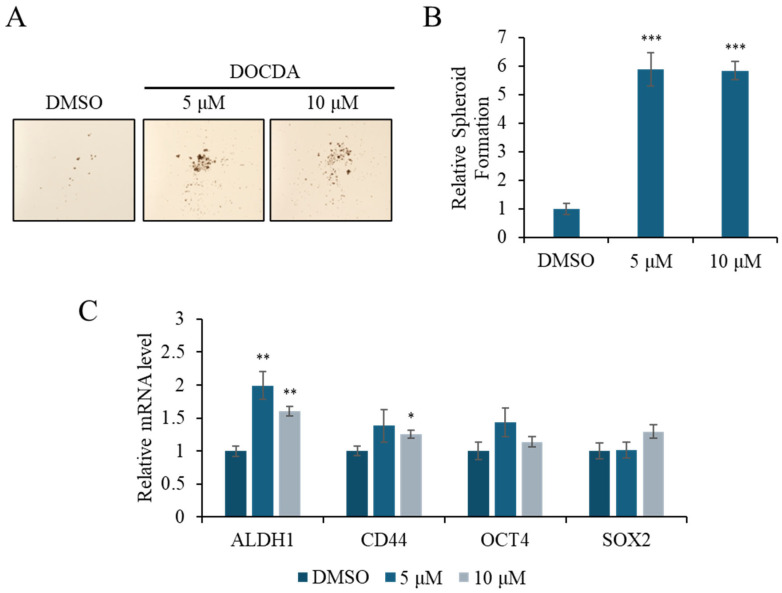
DOCDA treatment induces spheroid formation and modulates stemness-associated gene expression in HaCaT cells. (**A**,**B**) A spheroid formation assay was conducted using HaCaT cells treated with DOCDA (5 μM and 10 μM) or vehicle (DMSO) for 14 days. Quantitative analysis of spheroid size revealed a significant increase in spheroid-forming capacity upon DOCDA treatment. Data are expressed as mean spheroid area ± SD (*n* = 3); *** *p* < 0.001 vs. vehicle (DMSO). (**C**) qRT-PCR analysis of stemness-associated markers (ALDH1, CD44, OCT4, and SOX2) was performed on HaCaT cells treated with DOCDA. Relative mRNA levels were normalized to ACTIN and presented as fold changes compared to the DMSO control. Data are shown as mean ± SEM (*n* = 3); * *p*  <  0.05; ** *p*  <  0.01 vs. vehicle (DMSO).

Emerging evidence indicates that stemness-related pathways are critically involved in effective skin regeneration and wound repair [61]. Epidermal stem cells, particularly those expressing markers such as ALDH, CD44, OCT4, and SOX2, contribute to tissue repair by promoting self-renewal, differentiation, and enhanced cellular plasticity at the wound site [62,63,64,65,66,67]. ALDH is associated with epithelial cell proliferation and re-epithelialization, while CD44, a cell surface glycoprotein involved in adhesion and migration, plays a pivotal role in keratinocyte activation and matrix interaction [7,52,68,69,70]. To assess the impact of DOCDA on the stem-like properties of HaCaT cells, we performed a spheroid formation assay and analyzed the expression of stemness-related genes. DOCDA treatment markedly increased spheroid formation compared to the DMSO control. Specifically, both 5 μM and 10 μM concentrations significantly increased the average spheroid diameter, indicating an enhanced aggregation capacity (Figure 6A,B). In line with this phenotypic effect, qRT-PCR revealed a notable upregulation of several stemness markers. Treatment with 5 μM DOCDA induced an approximately 2-fold increase in ALDH1 expression, along with elevated CD44 and OCT4 mRNA levels. Notably, the transcription factor SOX2 also showed a modest but consistent increase, particularly at 10 μM (Figure 6C). These results suggest that DOCDA promotes wound healing by enhancing the expression of genes associated with cellular stemness and regenerative potential.

**Figure 7:**
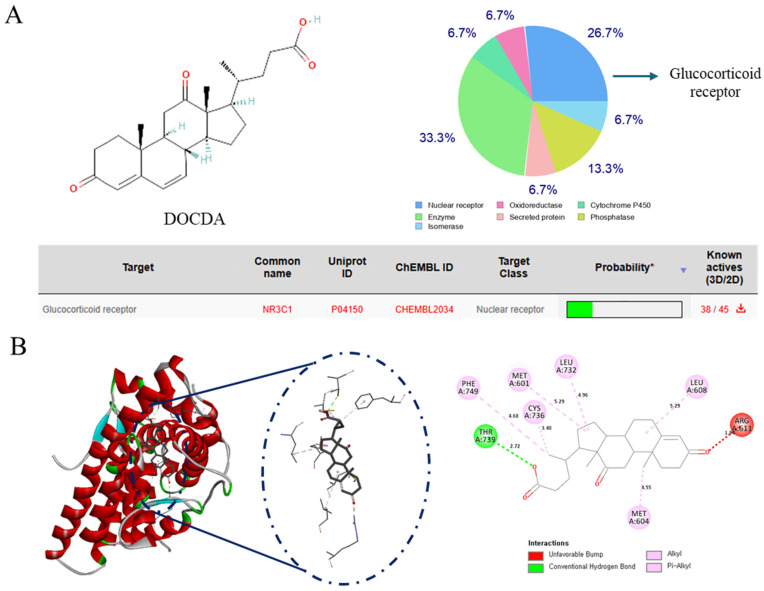
Identification of the GR as a potential target of DOCDA. (**A**) Chemical structure of DOCDA and results from SwissTargetPrediction analysis, identifying the glucocorticoid receptor (GR, also known as NR3C1) as the top predicted target. (**B**) Molecular docking results showing the binding interaction between DOCDA and GR (PDB ID: 4UDC), with a docking score of −7.7 kcal/mol. Key interacting residues are visualized in a 2D interaction diagram.

SwissTargetPrediction analysis indicated the GR as the most probable target of DOCDA. Molecular docking studies further supported this finding, demonstrating a strong binding affinity between DOCDA and GR, with a docking score of –7.7 kcal/mol. Docking was performed using the GR crystal structure (PDB ID: 4UDC). A 2D interaction diagram illustrates key interactions between DOCDA and specific GR residues. These results suggest that DOCDA may exert its biological activity via the modulation of GR, positioning it as a potential therapeutic target (Figure 7). In addition, hydrocortisone was used as a positive control in the molecular docking studies, showing a binding score of –9.6 kcal/mol (Figure S8).

To explore the molecular mechanisms underlying the wound healing effects of DOCDA, we performed an integrated analysis combining target prediction and disease relevance. Potential targets of DOCDA were identified using SwissTargetPrediction, while genes associated with “skin wound healing” were retrieved from GeneCards. A total of 67 overlapping genes were identified and analyzed through protein–protein interaction (PPI) network analysis. The resulting network revealed functional clustering centered around the GR, suggesting its pivotal role in mediating the effects of DOCDA. Subsequent pathway enrichment analysis using Metascape indicated that these genes are significantly enriched in pathways related to nuclear receptor activity, the regulation of hormone levels, inflammatory responses, steroid hormone metabolism, glucocorticoid biosynthesis, immune activation, cellular responses to estrogen stimuli, corticosteroid response, and mononuclear cell migration. The key enrichment of terms such as “nuclear receptors” and “response to corticosteroids” reinforces the potential involvement of GR as a key regulator of anti-inflammatory pathways and keratinocyte function.

**Figure 8:**
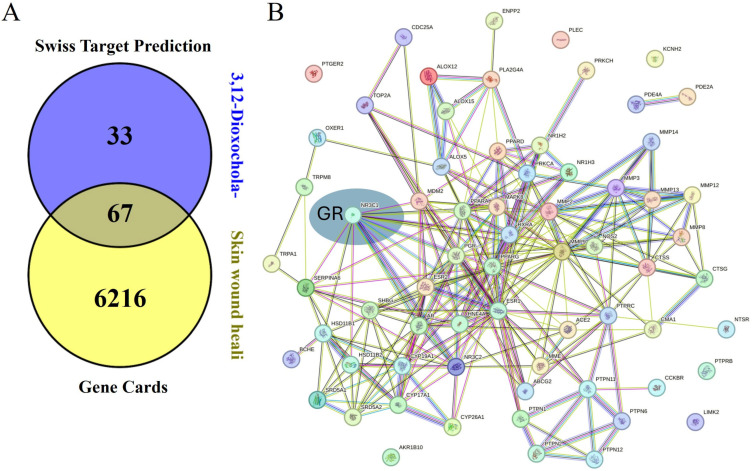

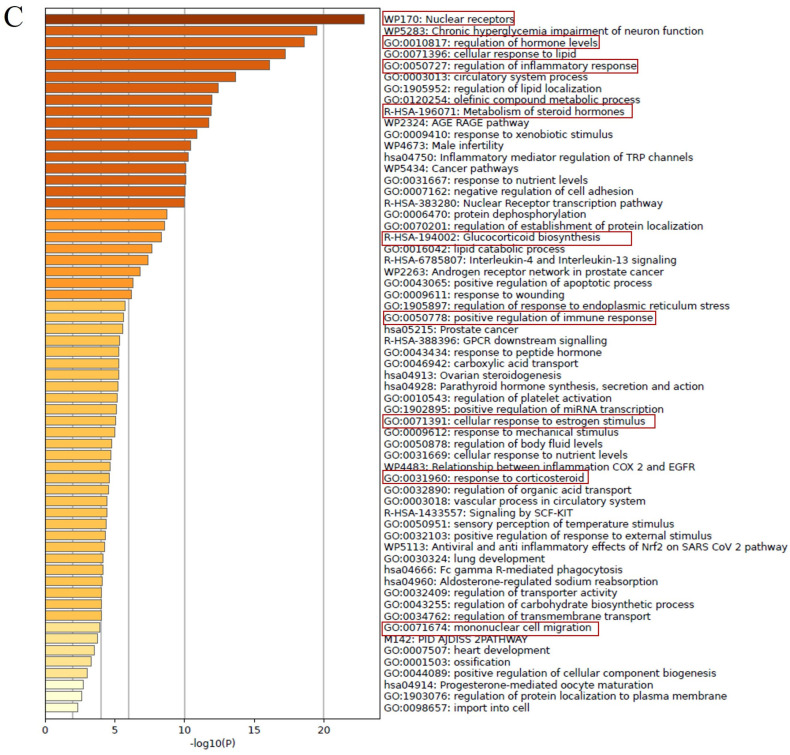
Network and pathway analysis of DOCDA-associated wound healing targets. (**A**) Potential targets of the steroid-like compound DOCDA were predicted using SwissTargetPrediction, and genes associated with the biological process “skin wound healing” were retrieved from GeneCards. (**B**) A total of 67 overlapping genes between predicted DOCDA targets and wound healing-associated genes were subjected to PPI analysis using the STRING database, revealing a functionally connected network centered around the GR. (**C**) Pathway enrichment analysis performed with Metascape demonstrated that these overlapping genes are significantly enriched in pathways relevant to wound healing, including nuclear receptors, regulation of hormone levels, regulation of inflammatory response, metabolism of steroid hormones, glucocorticoid biosynthesis, positive regulation of immune response, cellular response to estrogen stimulus, response to corticosteroids, and mononuclear cell migration.

Recent studies have shown that GR activation influences keratinocyte motility and proliferation during wound repair, underscoring its multifaceted role in tissue regeneration [71]. Moreover, enrichment in immune-related pathways, including the regulation of inflammatory and immune responses, reflects the critical balance between inflammation and immune activation necessary for proper wound healing. Estrogen signaling, also enriched in this analysis, is known to modulate genes involved in epidermal regeneration, inflammation, matrix remodeling, and protease inhibition, all of which are essential for effective wound repair [72]. Notably, estrogen has been shown to enhance angiogenesis, fibroblast proliferation, and extracellular matrix production. Studies have indicated that estrogen deprivation impairs wound healing, whereas topical estrogen application can reverse age-associated delays in wound repair in elderly men and women [73]. Additionally, the enrichment of “mononuclear cell migration” suggests a role in immune cell recruitment to the wound site, a key step in clearing cellular debris and initiating tissue repair. Recent studies have demonstrated the therapeutic potential of mononuclear cells in promoting wound closure and limb salvage [74]. Collectively, these findings support the hypothesis that DOCDA enhances wound healing through the modulation of steroid hormone signaling, immune responses, and GR-regulated cellular processes (Figure 8).

To assess the pharmacokinetic and toxicological potential of DOCDA as a novel wound healing agent, we conducted a comparative in silico ADMET analysis with hydrocortisone, a clinically established topical corticosteroid. In terms of absorption, both DOCDA and hydrocortisone were predicted to be bioavailable and well-absorbed in the human intestine, despite exhibiting a relatively low permeability in Caco-2 and MDCK models (DOCDA: −4.94 and −4.74 logPaap/cm/s; hydrocortisone: −4.55 and −4.6 logPaap/cm/s). These values indicate limited passive diffusion across intestinal membranes. Both compounds are also not substrates or inhibitors of P-glycoprotein (P-gp), reducing the likelihood of efflux-related absorption limitations. Notably, DOCDA demonstrated a better skin permeability (log Kp: −1.66) compared to hydrocortisone (−2.12), suggesting more efficient dermal absorption. Regarding distribution, both compounds were predicted to be blood–brain-barrier-permeable, although hydrocortisone showed a slightly lower penetration potential (log PS: −3.05 vs. −2.48). DOCDA exhibited higher plasma protein binding (84.06%) and a greater fraction unbound (1.37) than hydrocortisone (67.43% binding, 0.78 unbound), which may influence both distribution and pharmacodynamics. The volume of distribution at steady state (log VDss) was also higher for DOCDA (0.76) than hydrocortisone (0.47), suggesting a broader tissue distribution. In terms of metabolism, both compounds were identified as CYP3A4 substrates and showed no inhibitory activity against major CYP450 enzymes (CYP1A2, CYP2C19, CYP2C9, and CYP2D6). However, hydrocortisone was predicted to be a substrate for CYP2C19, which may impact its metabolic clearance. Importantly, both were predicted to inhibit OATP1B1, indicating a potential for hepatic-uptake-related drug–drug interactions, while showing no inhibitory effect on OATP1B3 or BCRP. Excretion parameters revealed that hydrocortisone had a higher predicted clearance (6.16 log ml/min/kg) compared to DOCDA (4.73), indicating potentially faster systemic elimination. Both compounds were estimated to have short half-lives (<3 h), suggesting the need for frequent dosing to maintain therapeutic levels. Notably, DOCDA does not inhibit the organic cation transporter OCT2, unlike hydrocortisone, which may reduce renal transporter-mediated interactions. Toxicological predictions highlighted some critical differences. DOCDA was non-mutagenic in the AMES, while hydrocortisone was predicted to be mutagenic. Both compounds showed endocrine-related toxicities via androgen receptor (NR-AR) interaction, but hydrocortisone was also predicted to be toxic at the AR-LBD, ER, and GR receptors—raising concerns about broader endocrine disruption. Interestingly, DOCDA exhibited the activation of the oxidative stress-related SR-ARE pathway, indicating potential for redox imbalance, whereas hydrocortisone did not. Both compounds were safe in terms of eye irritation and skin sensitization, and showed a similar environmental safety based on bioconcentration and fathead minnow toxicity data. In conclusion, both DOCDA and hydrocortisone present a favorable oral bioavailability and metabolic stability, with a low risk for major CYP450-mediated drug interactions. However, DOCDA offers advantages in terms of skin permeability, plasma protein binding, and a cleaner mutagenic profile. Nevertheless, potential redox-related toxicity and endocrine effects via androgen receptor activation warrant further investigation. These comparative data support the preclinical advancement of DOCDA, while highlighting the need for comprehensive in vitro and in vivo validation, particularly in the context of endocrine and oxidative safety (Table 1).

**Figure 9:**
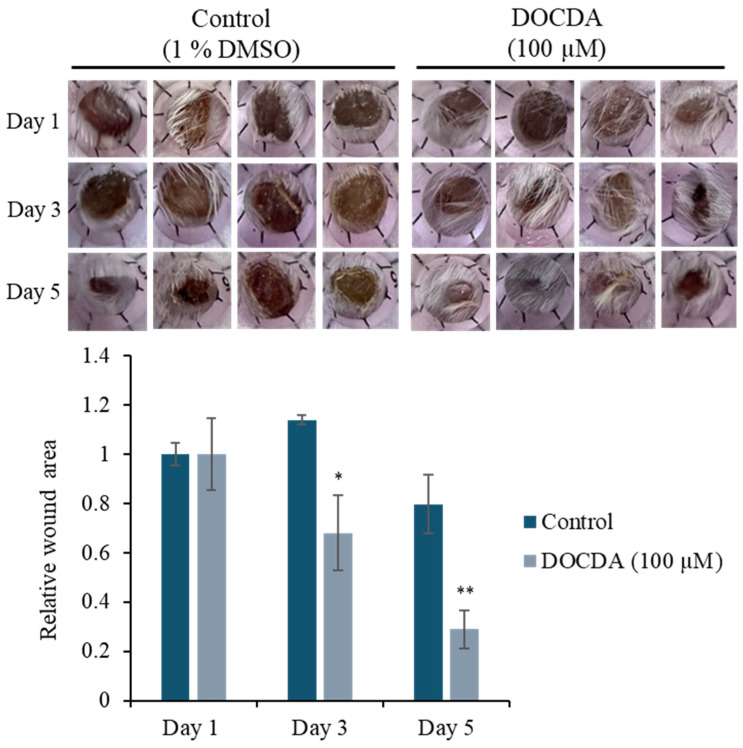
In vivo wound healing assay demonstrating enhanced wound closure with DOCDA treatment. Mice received topical DOCDA (100 μM) once daily, and wound areas were measured on days 1, 3, and 5 post-injury. Wound size is expressed relative to the area on day 1 (normalized to 1). DOCDA treatment significantly accelerated wound closure compared to the control group, with a marked reduction in wound area observed by day 5. Data are presented as mean ± SEM (*n* = 4); * *p* < 0.05, ** *p* < 0.01 vs. control.

To evaluate the therapeutic potential of DOCDA, we conducted an in vivo wound- healing assay over a five-day period. Wound closure was monitored on days 1, 3, and 5 post-treatment. The topical application of DOCDA (100 μM) significantly accelerated wound healing compared to the control group. By day 3, wounds treated with DOCDA had contracted to approximately 32% of their original area, while control wounds remained largely unhealed. Remarkably, by day 5, the DOCDA-treated group exhibited substantial wound closure, with ~71% of the wound area healed, whereas the control group retained ~80% of the original wound area, showing minimal healing. These findings suggest that DOCDA markedly promotes wound closure in vivo, supporting its potential as a pro-healing agent with therapeutic relevance for tissue regeneration (Figure 9).

This study presents promising findings regarding the wound healing efficacy of DOCDA; however, several limitations should be acknowledged. First, although a potential interaction between DOCDA and the glucocorticoid receptor (GR) is proposed, the mechanism of this remains speculative due to the lack of direct experimental validation. Specifically, functional assays such as GR knockdown, knockout models, and competitive binding studies were not included, which limits the mechanistic depth of our conclusions. Future studies incorporating these approaches will be necessary to confirm GR involvement. Second, the current in vivo evaluation was conducted exclusively in healthy murine models. While these provide valuable initial insights, they do not fully capture the complexity of chronic wound environments, such as those seen in diabetic or immunocompromised conditions. Studies using such clinically relevant models are essential for better translation to therapeutic applications in chronic wound management. Third, comparative analyses with conventional therapies would provide a more comprehensive understanding of DOCDA’s therapeutic value and positioning within the field. These limitations will be addressed in future investigations to strengthen the translational relevance and mechanistic understanding of DOCDA in wound healing.

## 3. Materials and Methods

### 3.1. General Experimental Procedures

NMR spectra were acquired using an Agilent 400-MR DD2 NMR spectrometer (Agilent Technologies, Santa Clara, CA, USA)) (^1^H at 400 MHz and ^13^C at 100 MHz in dimethyl sulfoxide-*d*_6_ (DMSO-*d*_6_) (Cambridge Isotope Laboratories (CIL), Inc., Tewksbury, MA, USA) at the Ewha Drug Development Research Core Center, and the signals of the residual solvent were used as an internal reference (*δ*_H_ 2.50 ppm and *δ*_C_ 39.5 ppm for DMSO). Low-resolution LC/MS analysis was conducted using a Waters Micromass ZQ LC/MS system (Waters Corp, Milford, MA, USA) equipped with a Phenomenex Luna C18(2) reversed-phase column (100 Å, 100 mm × 4.6 mm, 5 µm; Phenomenex, Torrance, CA, USA). An Agilent Technologies 1260 quadrupole LC system (Agilent Technologies, Santa Clara, CA, USA) was also used, under similar chromatographic conditions, with a flow rate of 1.0 mL/min. LC/MS experiments were carried out at the National Research Facilities and Equipment Center (NanoBioEnergy Materials Center), Ewha Womans University. Column chromatography (CC) was performed using reversed-phase C18 gel (70–230 mesh; Merck, Germany), eluted with a step-gradient solvent system of water (H_2_O) and methanol (MeOH). Fractions were further purified by reversed-phase HPLC using a Phenomenex Luna C18(2) column (100 Å, 250 mm × 10 mm, 5 µm).

### 3.2. Bacterial Isolation and Identification

A *Dendraster excentricus* (sand dollar) specimen was collected from Jeju Island, Republic of Korea. In total, 1 g of the sample was homogenized in 9 mL of sterile distilled water and spread onto modified SYP SW agar medium (10 g of starch, 4 g of yeast extract, 2 g of peptone, 34.75 g of sea salt, and 18 g of agar per 1 L of distilled water). The bacterial strain APA–142 was isolated on this medium. 16S rDNA sequencing identified APA–142 as being most closely related to *Rhodococcus qingshengii* JCM 15477, with a 99.9% sequence similarity. The 16S rDNA sequence of strain APA–142 was deposited in GenBank under accession number PV382396.1.

### 3.3. Cultivation, Extraction, and Isolation

The APA–142 strain was cultured in forty 2.5 L Ultra Yield Flasks, each containing 1 L of SYP SW medium (10 g of starch, 4 g of yeast extract, 2 g of peptone, and 34.75 g of sea salt in 1 L of distilled water). The cultures were incubated at 27 °C with shaking at 120 rpm for 7 days. Following cultivation, the culture broth (89 L in total) was extracted with ethyl acetate (EtOAc; 89 L). The organic layer was concentrated under reduced pressure using a rotary evaporator to yield 4.6 g of crude extract. The crude extract was then fractionated via flash column chromatography on C18 resin and eluted stepwise with distilled water and methanol mixtures (H_2_O/CH_3_OH = 80/20, 60/40, 50/50, 40/60, 30/70, 20/80, 0/100, 0/100, 400 mL each), resulting in the isolation of 8 fractions (F1–F8). Fraction 5 was further purified by reversed-phase HPLC using a Phenomenex Luna C18(2) column (250 × 100 mm, 5 μm, 100 Å) at a flow rate of 2.0 mL/min under isocratic conditions with 40% aqueous acetonitrile (CH_3_CN), monitored at 280 nm. This yielded 3,12-dioxochola-4,6-dien-24-oic acid (DOCDA; 4.8 mg, *t*_R_ = 50.0 min).

*3,12-Dioxochola-4,6-dien-24-oic acid (DOCDA)*: dark yellow oil, ^1^H NMR (400 MHz, DMSO-*d*_6_): *δ*_H_ 6.22 (dd, *J* = 2.5, 9.9 Hz, H-6), 6.19 (dd, *J* = 2.5, 9.9 Hz, H-7), 5.66 (s, H-4), 2.76 (t, *J* = 13.0 Hz, H-11*β*), 2.67 (t, *J* = 11.0 Hz, H-8*β*), 2.57 (m, H-2*α*), 2.28 (m, H-2*β*), 2.24 (t, *J* = 4.3 Hz, H-23*α*), 2.13 (quin, *J* = 4.3 Hz, H-23*β*), 2.01 (dd, *J* = 4.3, 12.8 Hz, H-11*α*), 1.89 (br q, H-15*α*), 1.89 (br q, H-16*α*), 1.89 (br q, H-17*α*), 1.81 (dddd, *J* = 2.0, 5.68, 13.4, H-1*α*), 1.73 (m, H-22*α*), 1.63 (m, H-1*β*), 1.50 (q, *J* = 13.8 Hz, H-15*β*), 1.45 (t, *J* = 4.0 Hz, H-10), 1.42 (br t, H-14*α*), 1.36 (d, *J* = 9.8 Hz H-16*β*), 1.28 (d, *J* = 7.7 Hz H-20*α*), 1.23 (t, *J* = 3.4 Hz H-22*β*), 1.15 (s, 3H-19*β*), 1.08 (s, 3H-18*β*), 0.78 (d, *J* = 6.4 Hz 3H-21); ^13^C NMR (100 MHz, DMSO-*d*_6_): *δ*_C_ 212.2 (C-12), 198.0 (C-3), 174.8 (C-24), 162.1 (C-5), 139.5 (C-7), 128.1 (C-6), 123.5 (C-4), 57.2 (C-13), 54.7 (C-14), 52.0 (C-9), 46.2 (C-17), 37.6 (C-11), 36.4 (C-10), 36.0 (C-8), 35.0 (C-20), 33.4 (C-2), 33.0 (C-1), 31.1 (C-23), 30.3 (C-22), 26.9 (C-16), 23.2 (C-15), 18.7 (C-21), 15.6 (C-19), 11.1 (C-18); LR-ESI-MS *m*/*z* = 385.23 [M + H]^+^.

### 3.4. Cell Culture

Human colorectal cancer cells (Caco-2), keratinocytes (HaCaT), and mouse embryonic fibroblast cells (NIH-3T3) were obtained from the American Type Culture Collection (ATCC) and maintained in either Roswell Park Memorial Institute (RPMI) 1640 medium or Dulbecco’s Modified Eagle Medium (DMEM) (GenDepot, Baker, TX, USA), supplemented with 10% fetal bovine serum and 1% penicillin–streptomycin. Cells were cultured at 37 °C in a humidified incubator with 5% CO_2_.

### 3.5. Cell Viability

The crude extract or compound was initially dissolved in dimethyl sulfoxide (DMSO; Sigma-Aldrich). NIH-3T3 and HaCaT cells were seeded in 96-well plates at a density of 3 × 10^3^ cells per well and allowed to adhere overnight. Following this incubation, the cells were treated with designated concentrations of the crude extract or compound for 48 h. After treatment, MTT reagent was added to each well, and the plates were incubated for 4 h under standard conditions (37 °C, 5% CO_2_). To solubilize the resulting formazan crystals, 150 μL of DMSO was added per well. Absorbance was then measured at 570 nm using a microplate reader (BioTek Instruments).

### 3.6. Invasion

Cell invasion was evaluated using Transwell chambers (Corning, Corning, NY, USA) fitted with polycarbonate membranes containing 8 μm pores, pre-coated with 1% (*w*/*v*) gelatin. The cells were suspended in medium containing 0.2% (*v*/*v*) bovine serum albumin and treated with the compound or DMSO (control) for 24 h. The lower chamber was filled with 600 µL of DMEM or RPMI medium supplemented with 0.2% (*v*/*v*) bovine serum albumin and 10 µg/mL of fibronectin as a chemoattractant. After 24 h of incubation, cells that had invaded through the membrane were fixed and stained using a Diff-Quik staining kit. Microscopic images of the invaded cells were captured, and quantification was performed using IMT iSolution software version 21.1 (IMT i-Solution Inc., Northampton, NJ, USA) [75].

### 3.7. Scratch Wound Healing Assay

HaCaT cells were seeded at a density of 4 × 10^4^ cells per well in 96-well ImageLock plates (Essen BioScience, Ann Arbor, MA, USA). After overnight incubation to allow for the formation of a confluent monolayer, uniform scratch wounds were created in all wells using the WoundMaker tool. Detached cells were removed by rinsing twice with serum-free DMEM, and the wells were replenished with DMEM supplemented with 2% (*v*/*v*) fetal bovine serum. The cells were then treated with either DOCDA or DMSO as a control. Wound closure was monitored using the IncuCyte imaging system equipped with a 10× objective lens. Images were acquired every 4 h taken per well under standard scanning settings. Cell migration was quantified as relative wound density, defined as the ratio of cell density within the wound area to that of the surrounding monolayer, using IncuCyte Software [76].

### 3.8. Spheroid Formation Assay

Cells were dissociated using trypsin and rinsed with DMEM/F12 medium supplemented with N2 (Invitrogen, Carlsbad, CA, USA). The medium was further enriched with human basic fibroblast growth factor (hbFGF; Invitrogen) and human recombinant epidermal growth factor (hrEGF; Biovision, Atlanta, GA, USA). The cells were plated in ultra-low attachment 24-well plates at a density of 5000 cells per well. After a 14-day incubation period, spheroid formation was evaluated using an inverted phase-contrast microscope. Sphere-forming capacity was assessed by quantifying the pixel intensity of randomly selected spheroid areas using IMT iSolution software version 21.1 (IMT iSolution Inc., Northampton, NJ, USA) [77].

### 3.9. qRT-PCR

Total RNA was extracted from HaCaT cells using RNAiso Plus (TaKaRa, Otsu, Japan). Complementary DNA (cDNA) was synthesized from 1 μg of RNA using M-MLV reverse transcriptase (Invitrogen, Carlsbad, CA, USA). Gene expression levels were quantified using SYBR Green-based reagents (Enzynomics, Seoul, Republic of Korea) [78].

### 3.10. Target Prediction and Networking Pharmacology

Potential targets of DOCDA were predicted using the SwissTargetPrediction web server (http://www.swisstargetprediction.ch, accessed on 16 March 2025), which employs a ligand-based approach. The SMILES representations of the compounds were obtained from PubChem [79]. To identify disease-related genes, the keyword “skin wound healing” was searched in the GeneCards database (https://www.genecards.org/, accessed on 16 March 2025). Overlapping targets between the compound-related and disease-related gene sets were identified using a Venn diagram generated with an online tool. These common target genes were then submitted to the STRING database (https://string-db.org/, accessed on 16 March 2025) for the construction of a PPI network. Subsequently, Gene Ontology (GO) and pathway enrichment analyses were performed using the Metascape platform (https://metascape.org/, accessed on 16 March 2025). Enriched terms related to immune response, inflammatory signaling, glucocorticoid biosynthesis, and wound repair were specifically highlighted [80].

### 3.11. Molecular Docking

To investigate the interaction between the ligand and the receptor binding site, the crystal structure of the GR protein (PDB ID: 4UDC) was retrieved from the Protein Data Bank, as referenced in previous studies [81]. The molecular docking of 3,12-Dioxochola-4,6-dien-24-oic acid and hydrocortisone was conducted using the CB-Dock web server (http://cao.labshare.cn/cb-dock/, accessed on 29 June 2025) [82]. Docking poses were analyzed and visualized in both 2D and 3D formats using BIOVIA Discovery Studio 2021. Binding scores were evaluated based on the data provided by CB-Dock.

### 3.12. In Silico Toxicity Assessment

The SMILES of the compounds were analyzed using the Deep-PK (https://biosig.lab.uq.edu.au/deeppk/prediction, accessed on 29 June 2025) web tool. Predictions of absorption, distribution, metabolism, elimination, and toxicity were obtained through deep learning-based models on the server. After downloading the prediction results, the basic ADMET parameters were evaluated.

### 3.13. Animal Model for Topical Wound Healing Evaluation

Animal experiments were conducted in accordance with previously reported protocols [25]. Male BALB/c mice (six weeks old, approximately 19–20 g) were obtained from Orientbio Inc. (Seongnam, Republic of Korea). All procedures were approved by the Institutional Animal Care and Use Committee of Sunchon National University and performed following ethical guidelines. Mice were housed individually in standard polycarbonate cages under controlled environmental conditions (temperature: 22 ± 2 °C; humidity: 50 ± 5%) with a 12 h light/dark cycle. Food and water were provided ad libitum. To induce full-thickness skin wounds, mice were anesthetized, and four circular excisional wounds (4 mm in diameter) were created on the dorsal skin using a disposable biopsy punch (Integra Miltex, Añasco, Puerto Rico). Each wound was topically treated with either a test compound or a control formulation. The test compound was initially dissolved in DMSO at a stock concentration of 10 mM and then diluted in a glycerol/PBS mixture (7:3, *v*/*v*) to a final concentration of 100 µM. The control treatment consisted of DMSO diluted in the same glycerol/PBS vehicle. A volume of 7 µL of each preparation was applied to the wounds 24 h after wound induction. Wound dimensions were measured prior to treatment and at designated time points thereafter. Both vertical and horizontal diameters were recorded, with all measurements performed by the same experimenter to ensure consistency.

### 3.14. Statistical Analysis

Statistical analyses were conducted using SigmaPlot version 12.5 (RRID:SCR_003210, Systat Software, Erkrath, Germany). Comparisons between two groups were performed using Student’s *t*-test. A *p*-value of ≤0.05 was considered statistically significant unless otherwise specified.

## 4. Conclusions

**Figure 10:**
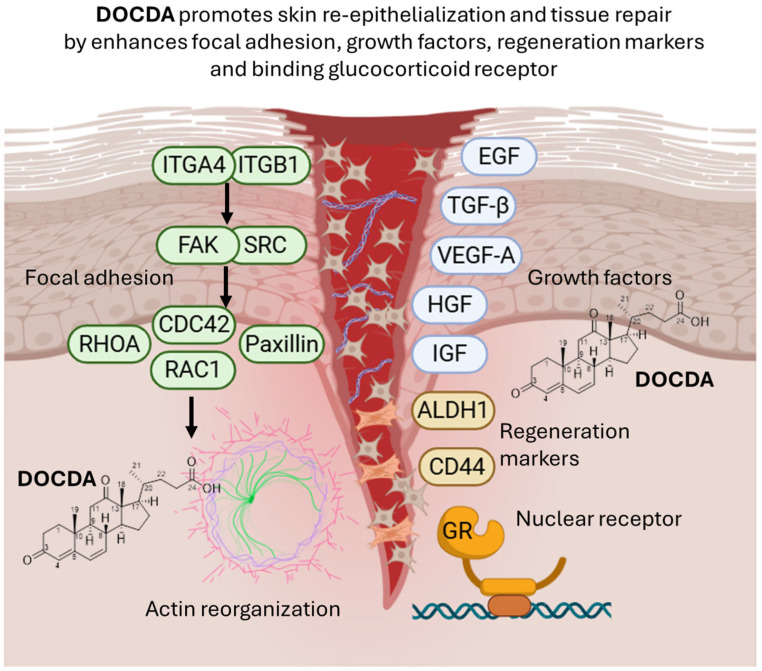
The diagram illustrates the role of DOCDA in promoting epithelial tissue regeneration. DOCDA modulate focal adhesion signaling by activating integrins (ITGA4 and ITGB1), focal adhesion kinase (FAK), and SRC, leading to downstream activation of RHOA, RAC1, and CDC42, which drive actin cytoskeleton reorganization. Paxillin also contributes to focal adhesion dynamics and actin remodeling. This cytoskeletal remodeling supports cell motility and tissue repair. Growth factors (EGF, TGF-*β*, VEGF-A, HGF, and IGF) are involved in enhancing the regenerative microenvironment. Regeneration markers ALDH1 and CD44 are upregulated in response to DOCDA, promoting stem-like cell behavior. Additionally, DOCDA binds the glucocorticoid receptor (GR), a nuclear receptor. Schematic representation created with BioRender.com (https://biorender.com, accessed on 29 June 2025).

This study demonstrates that DOCDA, a steroid-like compound derived from *Rhodococcus* sp. APA–142, enhances essential cellular processes involved in wound healing, including keratinocyte migration and proliferation. Mechanistic analyses indicate that DOCDA modulates pathways associated with focal adhesion dynamics, stemness markers, and growth factor signaling. In silico predictions further suggest a strong interaction between DOCDA and the GR, a central mediator of inflammation and tissue repair. Collectively, these findings suggest that DOCDA holds promising therapeutic potential for promoting tissue regeneration by targeting both cellular and molecular mechanisms critical for wound healing, as demonstrated through an integrated approach combining cell-based assays, animal models, and computational analyses (Figure 10).

## Figures and Tables

**Table 1:** Pharmacokinetic and toxicity properties of DOCDA and hydrocortisone.

PK and Toxicity Category	Property Name	Predicted Value		Property Unit
		DOCDA	Hydrocortisone	
**Absorption**	Caco-2	−4.94	−4.55	logPaap
	Human Oral Bioavailability 20%	Bioavailable	Bioavailable	Bioavailable/Non-Bioavailable
	Human Intestinal Absorption	Absorbed	Absorbed	Absorbed/Non-Absorbed
	Madin–Darby Canine Kidney	−4.74	−4.6	cm/s
	Human Oral Bioavailability 50%	Bioavailable	Bioavailable	Bioavailable/Non-Bioavailable
	P-Glycoprotein Inhibitor	Non-Inhibitor	Non-Inhibitor	Inhibitor/Non-Inhibitor
	P-Glycoprotein Substrate	Non-Substrate	Non-Substrate	Substrate/Non-Substrate
	Skin Permeability	−1.66	−2.12	log Kp
**Distribution**	Blood–Brain Barrier (Central Nervous System)	−2.48	−3.05	log PS
	Blood-Brain Barrier	Penetrable	Penetrable	Penetrating/Non-Penetrating
	Fraction Unbound (Human)	1.37	0.78	free proportion
	Plasma Protein Binding	84.06	67.43	therapeutic index
	Steady-State Volume of Distribution	0.76	0.47	log VDss
**Metabolism**	Breast Cancer Resistance Protein	Non-Inhibitor	Non-Inhibitor	Inhibitor/Non-Inhibitor
	CYP 1A2 Inhibitor	Non-Inhibitor	Non-Inhibitor	Inhibitor/Non-Inhibitor
	CYP 1A2_substrate	Non-Substrate	Non-Substrate	Substrate/Non-Substrate
	CYP 2C19 Inhibitor	Non-Inhibitor	Non-Inhibitor	Inhibitor/Non-Inhibitor
	CYP 2C19_substrate	Non-Substrate	Substrate	cyp2c19_substrate
	CYP 2C9 Inhibitor	Non-Inhibitor	Non-Inhibitor	Inhibitor/Non-Inhibitor
	CYP 2C9 Substrate	Non-Substrate	Non-Substrate	Substrate/Non-Substrate
	CYP 2D6 Inhibitor	Non-Inhibitor	Non-Inhibitor	Inhibitor/Non-Inhibitor
	CYP 2D6 Substrate	Non-Substrate	Non-Substrate	Substrate/Non-Substrate
	CYP 3A4 Inhibitor	Non-Inhibitor	Non-Inhibitor	Inhibitor/Non-Inhibitor
	CYP 3A4 Substrate	Substrate	Substrate	Substrate/Non-Substrate
	OATP1B1	Inhibitor	Inhibitor	Inhibitor/Non-Inhibitor
	OATP1B3	Non-Inhibitor	Non-Inhibitor	Inhibitor/Non-Inhibitor
**Excretion**	Clearance	4.73	6.16	Log (ml/min/kg)
	Organic Cation Transporter 2	Non-Inhibitor	Inhibitor	Inhibitor/Non-Inhibitor
	Half-Life of Drug	Half-Life < 3 h	Half-Life < 3 h	Half-life ≥ 3 h/Half-life < 3 h
**Toxicity**	AMES Mutagenesis	Safe	Toxic	Toxic/Safe
	Bioconcentration Factor	−0.61	−1.59	log10(L/kg)
	Biodegradation	Safe	Safe	Toxic/Safe
	Eye Corrosion	Safe	Safe	Toxic/Safe
	Eye irritation	Safe	Safe	Toxic/Safe
	Maximum Tolerated Dose	0.84	−0.15	log mg/kg/day
	NR-AhR	Safe	Safe	Toxic/Safe
	NR-AR	Toxic	Toxic	Toxic/Safe
	NR-AR-LBD	Safe	Toxic	Toxic/Safe
	NR-Aromatase	Safe	Safe	Toxic/Safe
	NR-ER	Safe	Toxic	Toxic/Safe
	NR-ER-LBD	Safe	Safe	Toxic/Safe
	NR-GR	Safe	Toxic	Toxic/Safe
	NR-PPAR-gamma	Safe	Safe	Toxic/Safe
	NR-TR	Safe	Safe	Toxic/Safe
	Rat (Acute)	2.2	1.95	log[1/(mol/kg)]
	Rat (Chronic Oral)	2.08	2.11	log(mg/kg_bw/day)
	Fathead Minnow	3.92	3.92	−log10 [(mg/L)/(1000 × MW)]
	Skin Sensitization	Safe	Safe	Toxic/Safe
	SR-ARE	Toxic	Safe	Toxic/Safe
	SR-ATAD5	Safe	Safe	Toxic/Safe
	SR-HSE	Safe	Safe	Toxic/Safe
	SR-MMP	Safe	Safe	Toxic/Safe
	SR-p53	Safe	Safe	Toxic/Safe

## Data Availability

The data for the research results can be obtained from Supporting Information.

## Supplementary Materials

The following supporting information can be downloaded at: https://www.mdpi.com/article/10.3390/md23070292/s1, Figure S1: ^1^H NMR spectrum (400 MHz) of DOCDA in DMSO; Figure S2: ^13^C NMR spectrum (100 MHz) of DOCDA in DMSO; Figure S3: COSY spectrum (400 MHz) of DOCDA in DMSO; Figure S4: HSQC spectrum (400 MHz) of DOCDA in DMSO; Figure S5: HMBC spectrum (400 MHz) of DOCDA in DMSO; Figure S6: NOESY spectrum (400 MHz) of DOCDA in DMSO; Figure S7: Effects of APA–142 fractions on the invasive capacity of Caco-2 and NIH3T3 cells. Transwell invasion assays were performed following 24 h treatment with various fractions of APA–142; Figure S8. Molecular docking results showing the binding interaction between hydrocortisone and GR (PDB ID: 4UDC), with a docking score of −9.6 kcal/mol. Key interacting residues are visualized in a 2D interaction diagram.

## Abbreviations

DOCDA	3,12-dioxochola-4,6-dien-24-oic acid
HaCaT	Human Adult Low Calcium Temperature Keratinocytes
EGF	Epidermal Growth Factor
VEGF-A	Vascular Endothelial Growth Factor A
IGF	Insulin-like Growth Factor
TGF-β	Transforming Growth Factor Beta
HGF	Hepatocyte Growth Factor
ITGB1	Integrin Subunit Beta 1
ITGA4	Integrin Subunit Alpha 4
FAK	Focal Adhesion Kinase
SRC	Proto-oncogene tyrosine-protein kinase Src
RHOA	Ras Homolog Family Member A
CDC42	Cell Division Cycle 42
RAC1	Ras-related C3 botulinum toxin substrate 1
GR	Glucocorticoid Receptor
ALDH1	Aldehyde Dehydrogenase 1
CD44	Cluster of Differentiation 44
