# Supplementary material for: A Marine-Derived Steroid from Rhodococcus sp., 3,12-Dioxochola-4,6-dien-24-oic Acid, Enhances Skin Re-Epithelialization and Tissue Repair
Source: Mar Drugs. 2025 Jul 19;23(7):292. doi: 10.3390/md23070292 (PMC12299624; doi:10.3390/md23070292)
Supplement: Supplementary file 1 [file marinedrugs-23-00292-s001.zip › marinedrugs-3726822-supplementary.pdf]

Supporting Information

A marine-derived steroid from *Rhodococcus* sp., 3,12-dioxochola-4,6-dien-24-oic acid, enhances skin re-epithelialization and tissue repair

Mücahit Varlı[1,†], Hui Tan[2,†], Chaeyoung Lee[2], Jeongyun Lee [1], Ji Young Lee[3], Jeong-Hyeon Kim[2], Songyi Lee[4,5], Hangun Kim[1,*] and Sang-Jip Nam[2,6,*]

[1] College of Pharmacy, Sunchon NationalUniversity, Sunchon, Jeonnam 57922, Republic of Korea

[2] Department of Chemistry and Nanoscience, Ewha Womans University, Seoul 03760, Republic of Korea

[3] Institute of Sustainable Earth and Environmental Dynamics (SEED), Pukyong National University, 365 Sinseon-ro, Nam-gu, Busan 48547, Republic of Korea

[4] Department of Chemistry, Pukyong National University, Busan 48513, Republic of Korea

[5] Industry 4.0 Convergence Bionics Engineering, Pukyong National University, Busan 48513, Republic of Korea

[6] Graduate Program in Innovative Biomaterials Convergence, Ewha Womans University, Seoul 03760, Republic of Korea

* Correspondence: hangunkim@scnu.ac.kr (H.G.K.), Tel.: +82-61-750-3761; sjnam@ewha.ac.kr (S.-J.N.), Tel.: +82-2-3277-6805

† These authors contributed equally to this work.

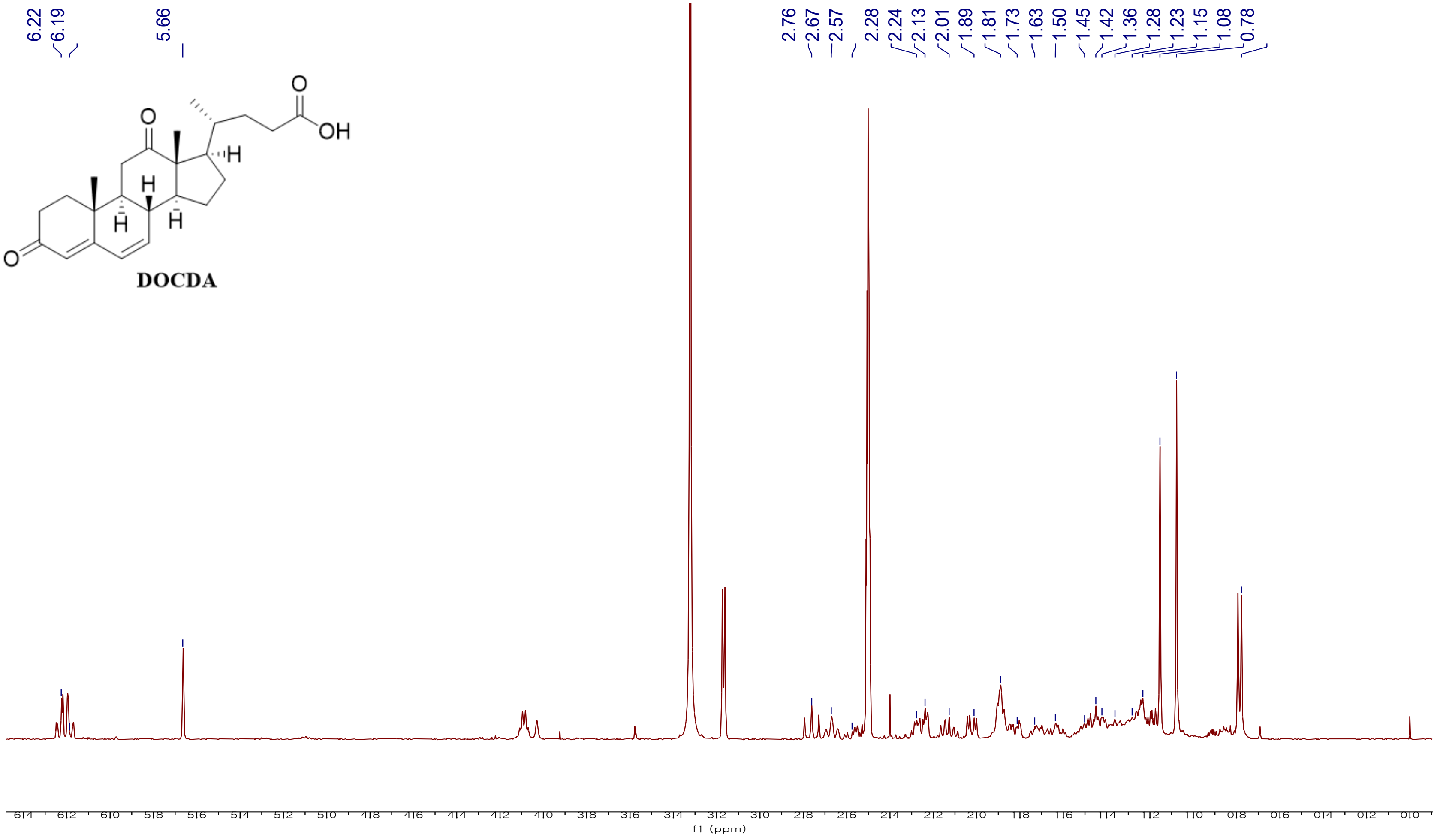

Figure S1. ¹H NMR spectrum (400 MHz) of DOCDA in DMSO.

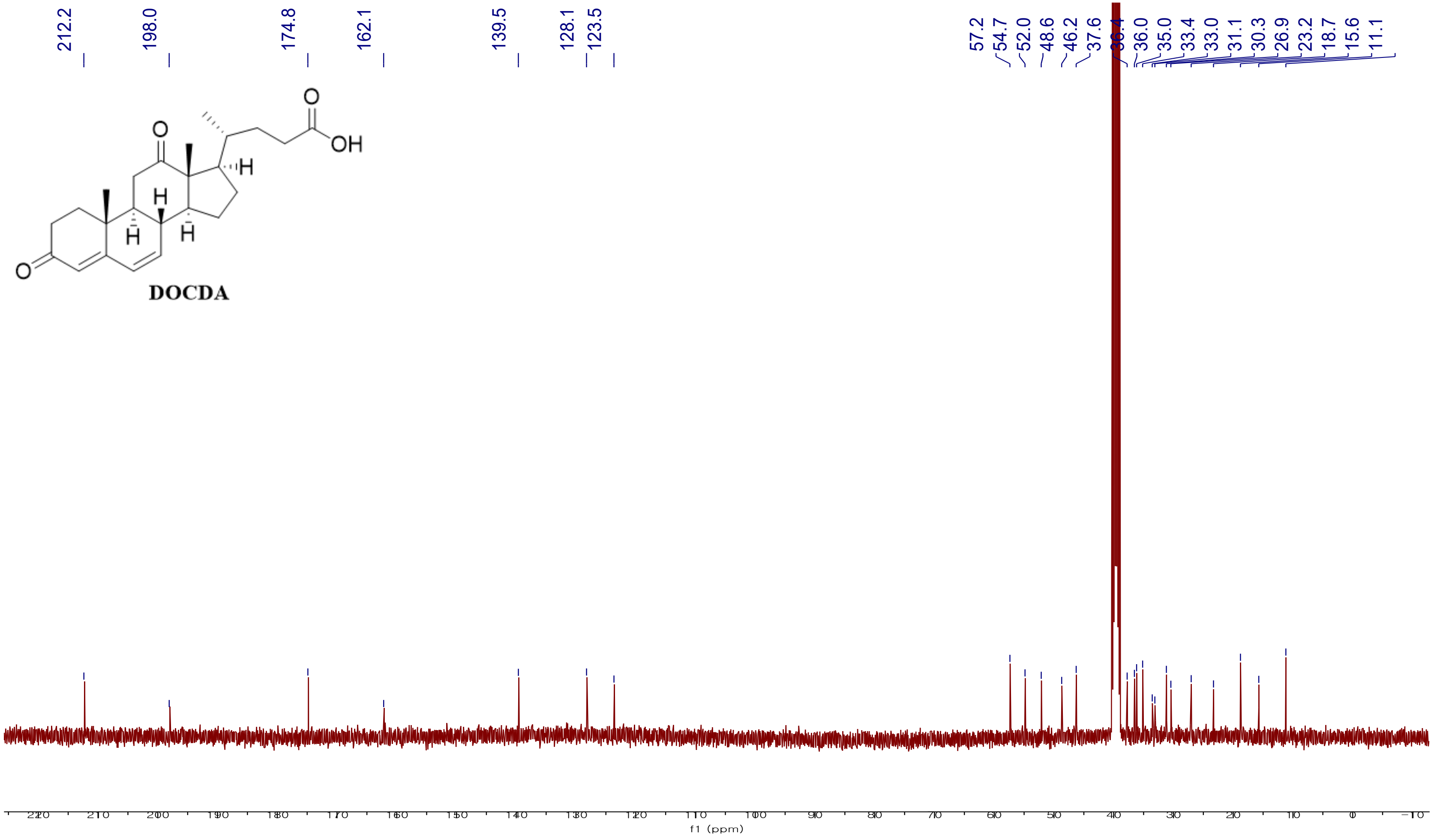

Figure S2. ^{13}C NMR spectrum DOCDA in DMSO.

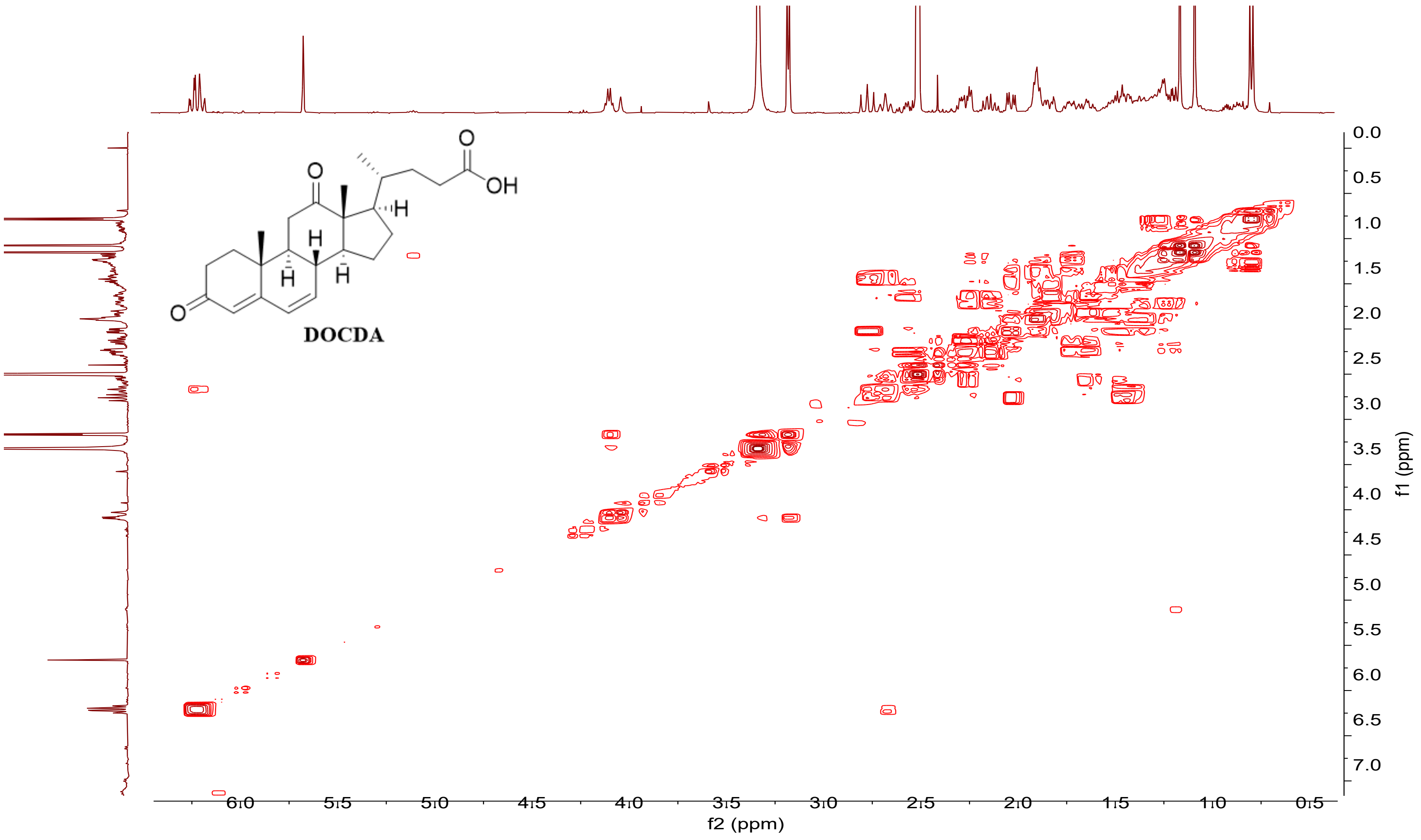

Figure S3. COSY spectrum (400 MHz) of DOCDA in DMSO.

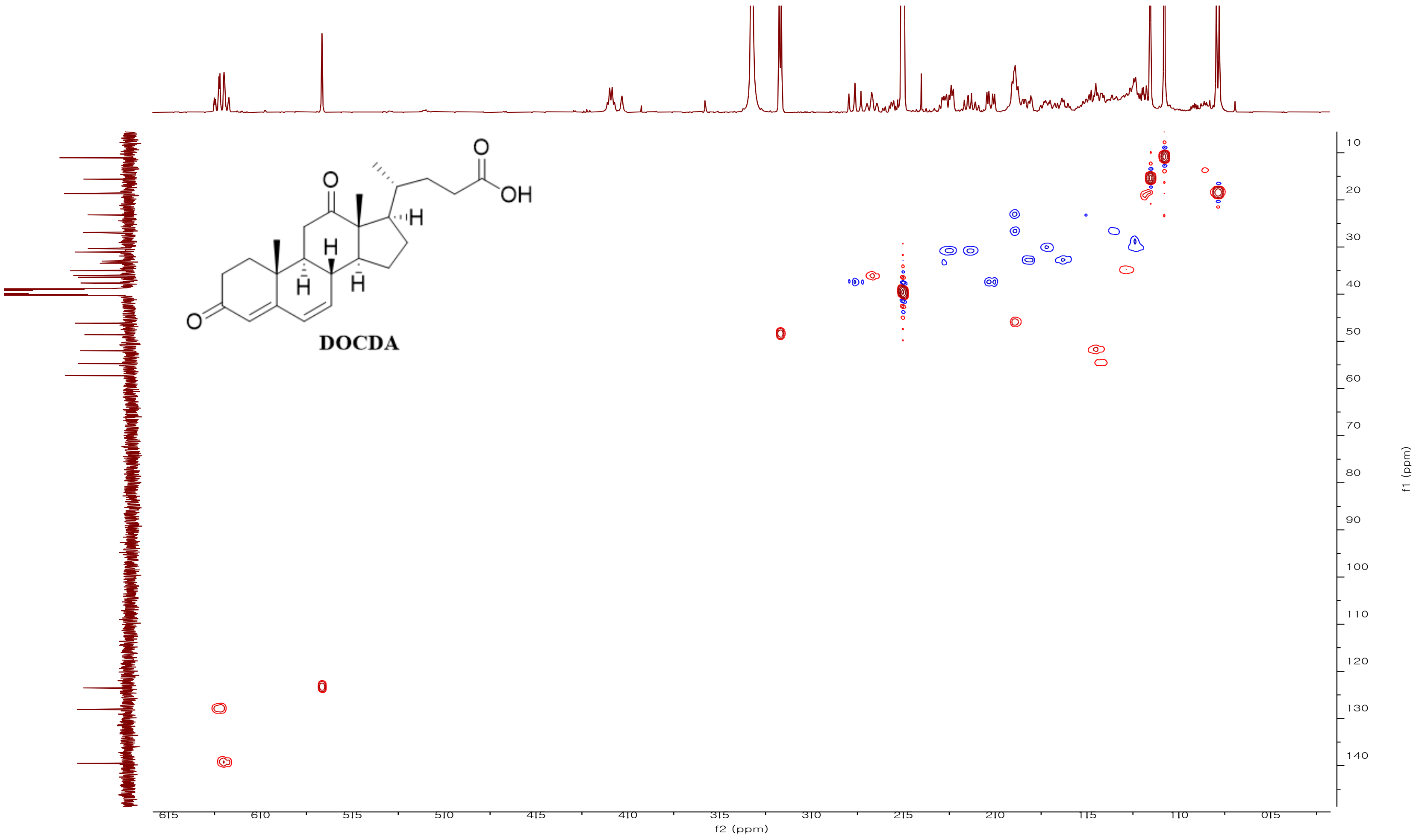

Figure S4. HSQC spectrum (400 MHz) of DOCDA in DMSO.

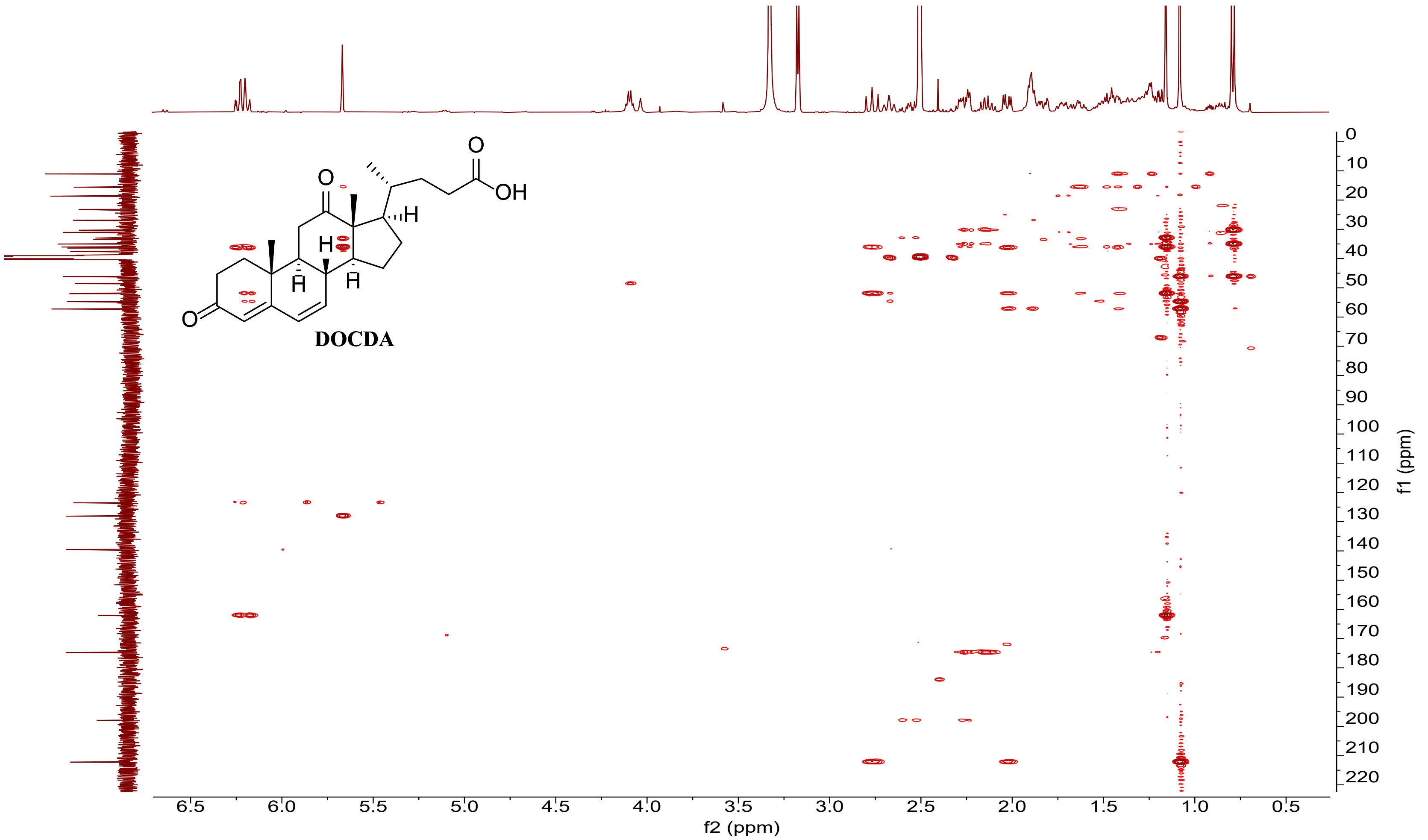

Figure S5. HMBC spectrum (400 MHz) of DOCDA in DMSO.

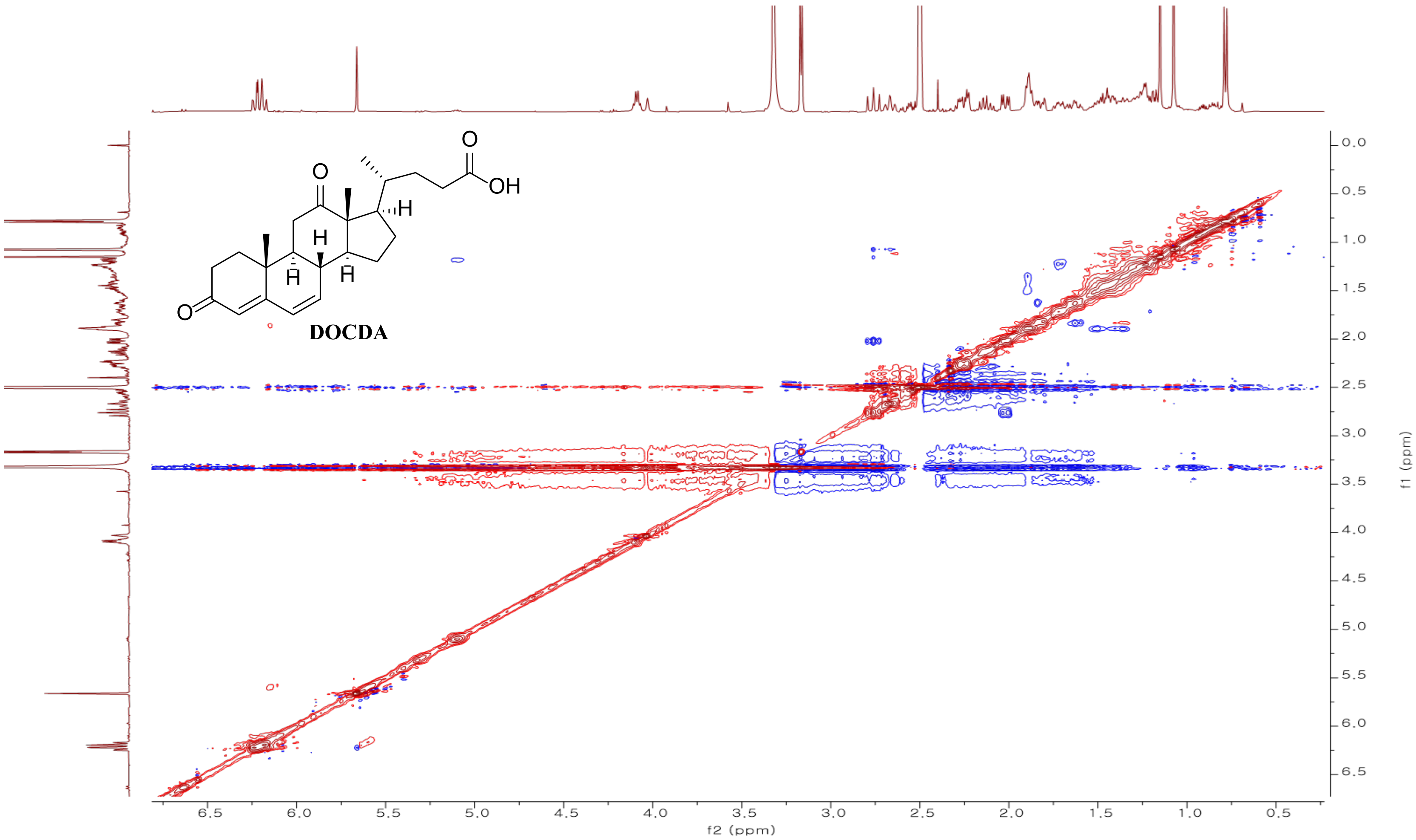

Figure S6. NOESY spectrum (400 MHz) of DOCDA in DMSO.

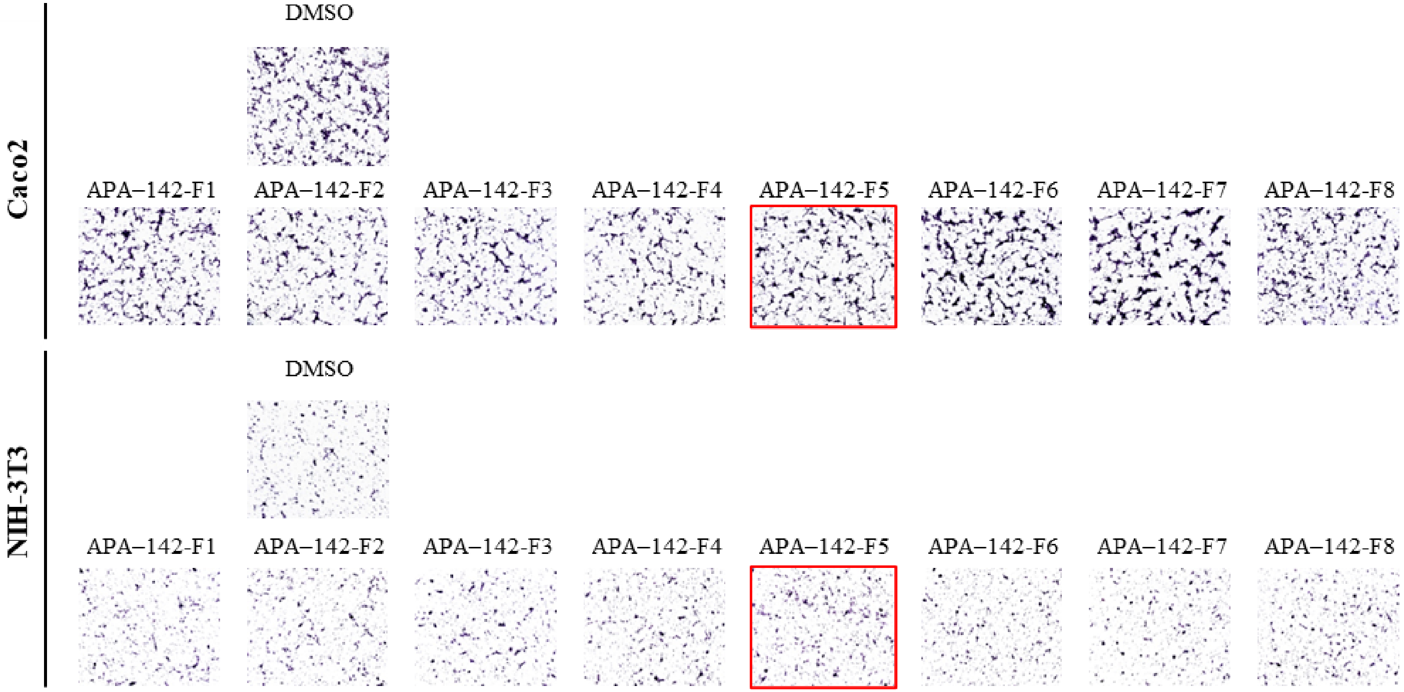

Figure S7. Effects of APA–142 fractions on the invasive capacity of Caco-2 and NIH3T3 cells. Transwell invasion assays were performed following 24-hour treatment with various fractions of APA–142.

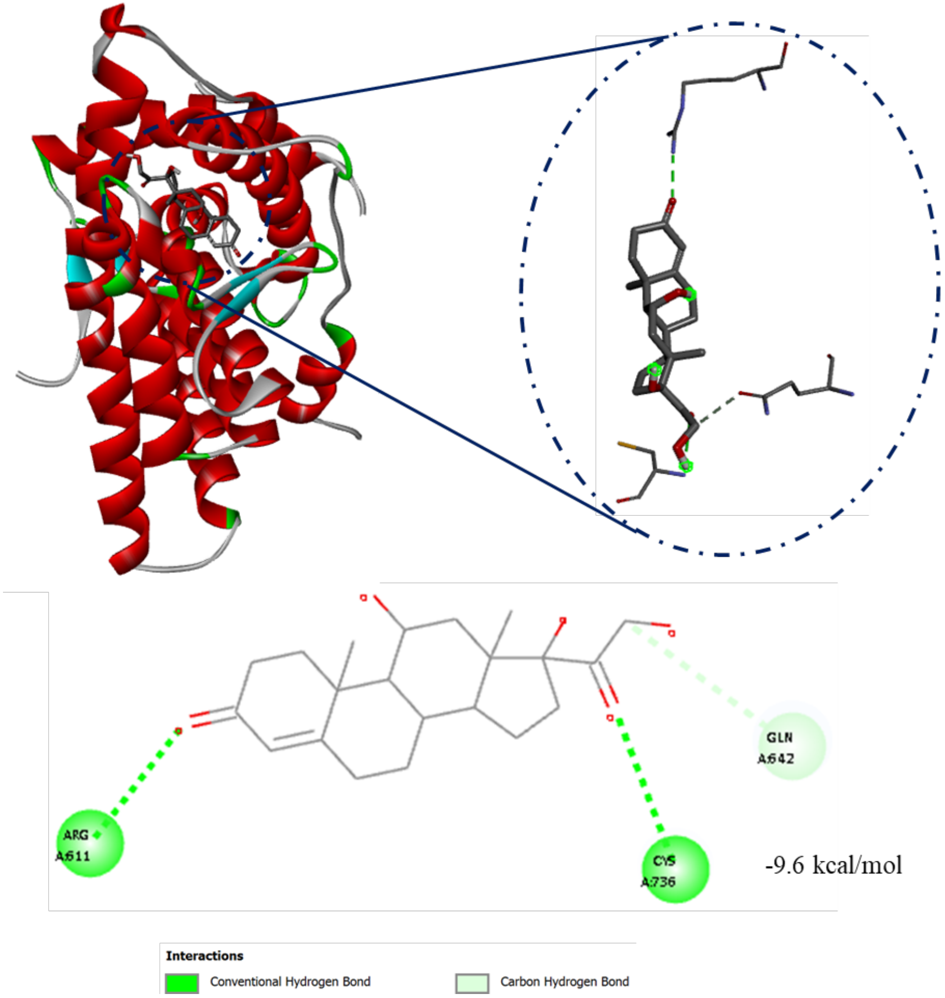

Figure S8. Molecular docking results showing the binding interaction between hydrocortisone and GR (PDB ID: 4UDC), with a docking score of –9.6 kcal/mol. Key interacting residues are visualized in a 2D interaction diagram.
